# Supplementary figures and images for: Implantable cardioverter defibrillator therapy is cost effective for primary prevention patients in Taiwan: An analysis from the Improve SCA trial
Source: PLoS One. 2020 Nov 19;15(11):e0241697. doi: 10.1371/journal.pone.0241697 (PMC7676667; doi:10.1371/journal.pone.0241697)

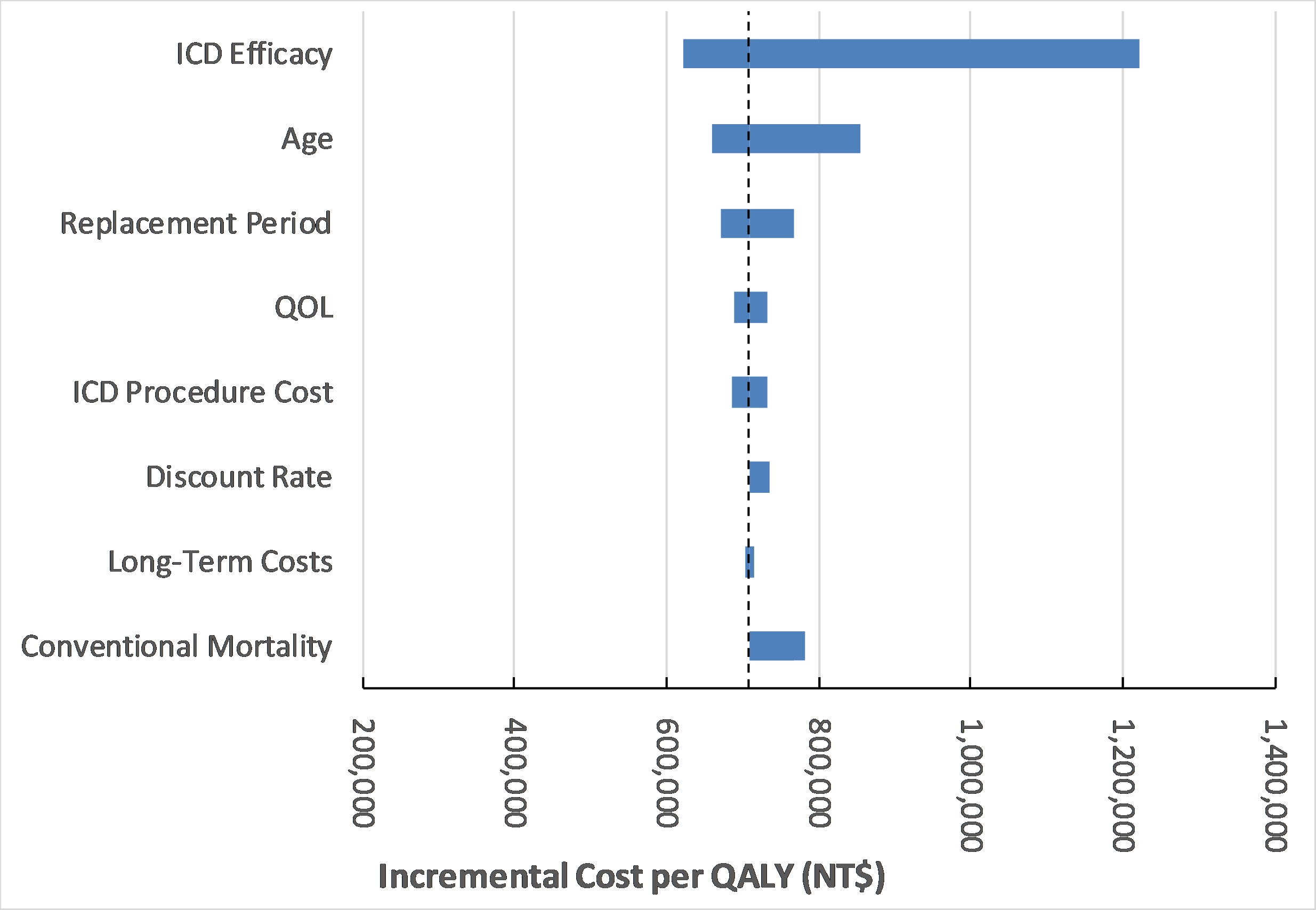

Supplement: S1 Fig — (TIF) [file pone.0241697.s001.tif]

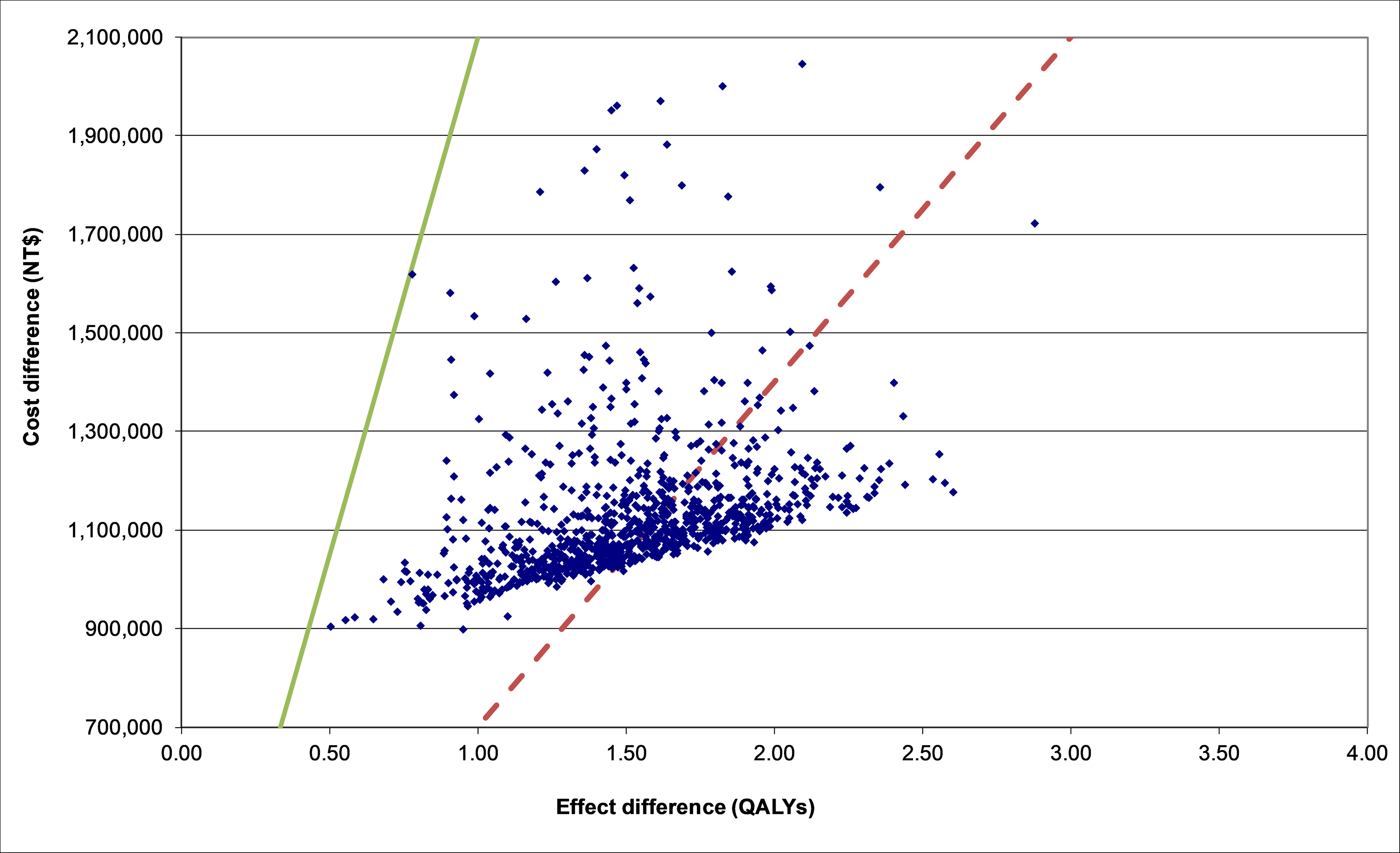

Supplement: S2 Fig — (TIF) [file pone.0241697.s002.tif]
