# Supplementary material for: Implantable cardioverter defibrillator therapy is cost effective for primary prevention patients in Taiwan: An analysis from the Improve SCA trial
Source: PLoS One. 2020 Nov 19;15(11):e0241697. doi: 10.1371/journal.pone.0241697 (PMC7676667; doi:10.1371/journal.pone.0241697)
Supplement: S1 Table — (DOCX) [file pone.0241697.s003.docx]

**S1 Table. Characteristics and result of economic evaluations of ICD for primary prevention**

| References | Country | Design | Comparators | Population | Perspective | Time horizon | Discount of costs and benefits | Outcome measure | Costs included | ICER | Acceptable CE thresholds in publication |
| --- | --- | --- | --- | --- | --- | --- | --- | --- | --- | --- | --- |
| (1) | USA | CEA  Markov model | ICD vs. amiodarone | MI without symptomatic, sustained VT | Societal | Lifetime | 3% | LY and QALY | DMC  DNMC | LVEF $\leq$ 30%:  US$63,300/LY; US$71,800/QALY  LVEF = 31%-40%: US$173,400/LY  US$195,700/QALY | US$75,000 |
| (2) | USA | CEA  Markov model | ICD vs. BMT | Several types of patients according to different RCT (MADIT, CABG-Patch, MUSTT, MADIT II, DEFINITE, DINAMIT, SCD-HeFT) | Societal | Lifetime | 3% | LY and  QALY | DMC | SCD-HeFT:  US$50,700/LY  US$70,200/QALY  DEFINITE:  US$36,800/LY  US$51,300/QALY  MADIT:  US$25,300/LY  US$34,900/QALY  MADIT II:  US$39,000/LY  US$54,100/QALY  DINAMIT: ICD is dominated by BMT | US$100,000 |
| (3) | USA | CEA  Markov model | ICD vs. BMT ICD vs. ICD for selected patients | Ischemic heart disease and LVEF $\leq$ 0.30 | Societal | Lifetime | 3% | LY and  QALY | DMC | US$55,800/QALY  US$88,700/QALY | US$50,000 |
| (4) | Belgium | CEA  Markov model | ICD + BMT vs. BMT | Chronic HF, NYHA class II or III, or prior MI with or without HF. LVEF $\leq$ 0.35 | Healthcare  system | Lifetime | 3 % costs and  1.5 % benefits | LY and QALY | DMC | 24,751 €/LY  29,530 €/QALY (19,760–61,316) | € 60,000 |
| (5) | Germany | CEA Markov model | ICD + BMT vs. BMT | MADIT II patient (see Zwanziger) | Statutory  health  insurance | Lifetime | 3% | LY and QALY | DMC | 33,105 €/LY  44,736 €/QALY | ICER of ICD implt. = ICER of conv. therapy |
| (6) | The Netherlands | CUA  Markov model | ICD vs. No ICD | Patients included ischaemic and non-ischaemic heart disease. LVEF $\leq$ 0.40 | Societal | Lifetime | 4 % costs and 1.5 % benefits | QALY | DMC | 43,993 €/QALY | € 80,000 |
| (7) | Brazil | CEA  Markov model | ICD + BMT vs. BMT | HF, NYHA class II and III, LVEF $\leq$ 0.35 | Public healthcare system  Private system | 20 years | 3% | LY and QALY | DMC | Public healthcare system：  R$60,121/LY  R$68,318/QALY  Private system：  R$80,029/LY  R$90,942/QALY | R$ 37,311 |
| This study | Taiwan | CEA  Markov model | ICD + BMT vs. BMT | Chronic HF, NYHA class II or III, or prior MI with or without HF. LVEF $\leq$ 0.35 | Healthcare  system | Lifetime | 1.375% | LY and  QALY | DMC | Primary prevention:  NT$708,711/QALY  1.5 primary prevention:  NT$441,153/QALY | NT$2,234,520  (US$74,484) |

MADIT, CABG-Patch, MUSTT, MADIT II, DEFINITE, DINAMIT, SCD-HeFT are clinical trials

BMT best medical treatment, CEA cost-effectiveness analysis, CUA cost-utility analysis, DMC direct medical costs, HF heart failure, ICD implantable cardioverter defibrillator, LVEF left ventricular ejection fraction, LY life years, MI myocardial infarction, NYHA New York Heart Association, QALY quality adjusted life years, RCT randomized clinical trial, VT ventricular tachycardia, WTP willingness to pay #Sources: International Monetary Fund World Economic Outlook (October – 2019)

**S1 table references**

1. Sanders GD, Hlatky MA, Every NR, McDonald KM, Heidenreich PA, Parsons LS, et al. Potential cost-effectiveness of prophylactic use of the implantable cardioverter defibrillator or amiodarone after myocardial infarction. Ann Intern Med. 2001;135(10):870-83.

2. Sanders GD, Hlatky MA, Owens DK. Cost-effectiveness of implantable cardioverter-defibrillators. N Engl J Med. 2005;353(14):1471-80.

3. Chan PS, Stein K, Chow T, Fendrick M, Bigger JT, Vijan S. Cost-effectiveness of a microvolt T-wave alternans screening strategy for implantable cardioverter-defibrillator placement in the MADIT-II-eligible population. J Am Coll Cardiol. 2006;48(1):112-21.

4. Cowie MR, Marshall D, Drummond M, Ferko N, Maschio M, Ekman M, et al. Lifetime cost-effectiveness of prophylactic implantation of a cardioverter defibrillator in patients with reduced left ventricular systolic function: results of Markov modelling in a European population. Europace. 2009;11(6):716-26.

5. Gandjour A, Holler A, Dipl Ges O, Adarkwah CC. Cost-effectiveness of implantable defibrillators after myocardial infarction based on 8-year follow-up data (MADIT II). Value Health. 2011;14(6):812-7.

6. Smith T, Jordaens L, Theuns DA, van Dessel PF, Wilde AA, Hunink MG. The cost-effectiveness of primary prophylactic implantable defibrillator therapy in patients with ischaemic or non-ischaemic heart disease: a European analysis. Eur Heart J. 2013;34(3):211-9.

7. Ribeiro RA, Stella SF, Camey SA, Zimerman LI, Pimentel M, Rohde LE, et al. Cost-effectiveness of implantable cardioverter-defibrillators in Brazil: primary prevention analysis in the public sector. Value Health. 2010;13(2):160-8.
