# Supplementary material for: Implantable cardioverter defibrillator therapy is cost effective for primary prevention patients in Taiwan: An analysis from the Improve SCA trial
Source: PLoS One. 2020 Nov 19;15(11):e0241697. doi: 10.1371/journal.pone.0241697 (PMC7676667; doi:10.1371/journal.pone.0241697)
Supplement: S2 Table — (DOCX) [file pone.0241697.s004.docx]

**S2 Table. Taiwan utility analysis results.**

| **Contrast Estimate Results** | | | | | | | | |
| --- | --- | --- | --- | --- | --- | --- | --- | --- |
| Label | Mean Estimate | L’Beta Estimate | Standard Error | Alpha | L’Beta Confidence Limits | | Chi-Square | Pr > ChiSq |
| Mean Utility* | 0.7315 | 0.7315 | 0.0126 | 0.05 | 0.7067 | 0.7562 | 3359.3 | < 0.0001 |

*Mean Utility represents the utility calculated by applying Taiwan specific utility weights to the PAINFREE SST study EQ-5D quality of life data.(1)

1. Sears SF, Rosman L, Sasaki S, Kondo Y, Sterns LD, Schloss EJ, et al. Defibrillator shocks and their effect on objective and subjective patient outcomes: Results of the PainFree SST clinical trial. Heart Rhythm. 2018;15(5):734-40.
