# Supplementary material for: Implantable cardioverter defibrillator therapy is cost effective for primary prevention patients in Taiwan: An analysis from the Improve SCA trial
Source: PLoS One. 2020 Nov 19;15(11):e0241697. doi: 10.1371/journal.pone.0241697 (PMC7676667; doi:10.1371/journal.pone.0241697)
Supplement: Attachment — Submitted filename: Response to Reviewers 31 Aug 2020. .docx [file pone.0241697.s007.docx]

Response to reviewers, 31 Aug 2020.

Revised Manuscript. PONE-D-20-18597

**Manuscript Title:** Implantable cardioverter defibrillator therapy is cost effective for primary prevention patients in Taiwan: an analysis from the Improve SCA trial

*Dear PLOS ONE Reviews,*

*Thank you for your expeditious and thoughtful review. Please read below each question of your review for my responses, in red text.*

PONE-D-20-18597R1
Implantable cardioverter defibrillator therapy is cost effective for primary prevention patients in Taiwan: an analysis from the Improve SCA trial
Dr. Yen-Bin Liu

Dear Dr. Liu,

Thank you for submitting your manuscript entitled "Implantable cardioverter defibrillator therapy is cost effective for primary prevention patients in Taiwan: an analysis from the Improve SCA trial" to PLOS ONE.  Your manuscript files have been checked in-house but before we can proceed we need you to address the following issues:

1) Please amend your current ethics statement to include the full name of the ethics committee/institutional review board(s) that approved your specific study.

This analysis is a modeling exercise based on previously published data in the Improve SCA study (DOI: 10.1111/pace.13526) and does not involve any additional human research. The Improve SCA protocol was approved by the Institutional Review Board or Medical Ethics Committee of each respective study center (which are included in the Improve SCA study supplemental data). This information has been included in the revised cover letter ethics statement.

2) Please amend your current ethics statement to confirm that your named institutional review board or ethics committee specifically approved this study.

This analysis is a modeling exercise based on previously published data in the Improve SCA study (DOI: 10.1111/pace.13526) and does not involve any additional human research. Their protocol was approved by the Institutional Review Board or Medical Ethics Committee of each respective study. This information has been included in the revised cover letter ethics statement.

3) Once you have amended this/these statement(s) in the Methods section of the manuscript, please add the same text to the “Ethics Statement” field of the submission form (via “Edit Submission”).
For additional information about PLOS ONE ethical requirements for human subjects research, please refer to [http://journals.plos.org/plosone/s/submission-guidelines#loc-human-subjects-research](https://urldefense.com/v3/__http:/journals.plos.org/plosone/s/submission-guidelines*loc-human-subjects-research__;Iw!!NFcUtLLUcw!F2t3HAEYXFcmurQ8d2r7m5KPPk24qOhPESDn1o27m2TensL0Cusvm0Du23bz3o6vjNU$)

The following statement has been included in the Methods section of the manuscript, “The Improve SCA study protocol was approved by the Institutional Review Board or Medical Ethics Committee of each respective study center. This analysis is a modeling exercise based on previously published data from the Improve SCA study and does not involve any additional human research.” This has been included in the revised cover letter ethics statement.

4) Please include a copy of Table 2 which you refer to in your text in line 168.
Table 2 has been moved to immediately follow the sentence where it is first mentioned in the manuscript. A separate file o has of Table 2 has also been submitted.

*Thank you for your time and consideration.*

*Sincerely,*

*Dr Yen-Ben Liu*
